# No negative effect of mentioning COVID-19 vaccine in influenza vaccine encouragements: Evidence from a survey experiment

**DOI:** 10.1371/journal.pgph.0005180

**Published:** 2025-09-10

**Authors:** Filip Viskupič, David L. Wiltse

**Affiliations:** 1 Department of Political Science, Iowa State University of Science and Technology, Ames, Iowa, United States of America; 2 School of American and Global Studies, South Dakota State University, Brookings, South Dakota, United States of America; PLOS: Public Library of Science, UNITED STATES OF AMERICA

## Abstract

It is possible that the negative attitudes toward COVID-19 vaccination developed by some people, such as self-identified Republicans, might spill over toward other vaccines. We conducted a survey experiment to investigate if mentioning COVID-19 vaccine in messages encouraging seasonal flu vaccination will negatively affect people’s attitudes toward receiving a flu vaccine. The experiment was embedded in a survey fielded in South Dakota in April 2024. Participants were registered voters in the state. We used difference-of-means tests and logistic regression to analyze the data. Results showed no statistically significant differences between the participants in control and treatment groups regarding the likelihood of receiving a flu vaccine (OR: 1.012, CI: 0.594 - 1.724) or their belief in the safety of the flu vaccine (OR: 1.333, CI: 0.707- 2.511). We observed the same patterns when we only looked at Republicans. Attitudes toward flu vaccination were primarily driven by peoples’ current flu vaccination status. We found that mentioning COVID-19 vaccines in flu vaccination messages did not negatively affect people’s attitudes toward flu vaccine. Public health workers and officials might not need to shy away from mentioning COVID-19 vaccines when communicating other vaccines.

## Introduction

Seasonal influenza is a common respiratory infection. The Centers for Disease Control and Prevention (CDC) estimated that during the 2023–2024 flu season, there were 34–63 million flu illnesses, 16–29 million flu-related medical visits, 380,000–790,000 flu hospitalizations, and 24,000–69,000 flu deaths in the United States [1]. Flu vaccines are highly effective in preventing millions of symptomatic illness cases, hospital visits, and thousands of deaths each year [1,2]. However, according to CDC estimates, fewer than half of eligible adults received a flu vaccination during the 2023–2024 vaccination season [3].

While reaching a high flu vaccination rate was challenging even before the outbreak of COVID-19 in 2020, the pandemic created additional barriers to flu vaccination. COVID-19 vaccination quickly became a highly political and emotionally charged issue. Misinformation about the safety and effectiveness of COVID-19 vaccines abounded, particularly on social media [4–6]. Ultimately, attitudes toward COVID-19 became significantly structured on partisan self-identification. Scholars took note of how quickly COVID-19 vaccination became a politically polarizing issue at the elite and mass levels in several democracies around the world [7]. In the United States, politicians from the Democratic and Republican parties differed markedly in their public messaging toward the safety and effectiveness of COVID-19 vaccines [8–10]. Due to this elite polarization, the public discussion of COVID-19 was sharply split along partisan lines. Studies based on self-reported surveys showed lower vaccination rates among self-identified Republicans compared to Democrats [11–14]. Research also showed that Republicans were more likely to endorse anti-vaccine misinformation during the COVID-19 pandemic [15,16]. Additionally, Republican states reported more COVID-19 vaccination adverse events compared to Democratic states [17].

Scholars warned that the cold feelings toward COVID-19 vaccination could translate into hesitancy to receive other vaccines in what is commonly referred to as the “spillover effect” [18–20]. Studies conducted before the outbreak of COVID-19 showed that attitudes toward one vaccine were linked to attitudes toward other vaccines [21], suggesting that negative spillover might be possible in the COVID-19 era. The spillover will likely be the greatest among the groups with heightened negative feelings toward COVID-19 vaccines; for example, Republican Party supporters [22]. If true, the spillover of negative COVID-19 vaccine attitudes onto other vaccines could have major consequences for public health. This spillover has been found to affect other areas of life, far removed from peoples’ own health decisions. For example, scholars provided evidence that people who did not receive a COVID-19 vaccination were less likely to vaccinate their pets [23,24].

The development of negative feelings toward COVID-19 vaccines could undermine the objective of securing a high flu vaccination rate annually. Given the prevalence of the flu virus during the winter season, a lowered vaccination rate would have negative consequences for our society and economy. Besides the burden of morbidity and mortality, experts estimated that seasonal flu leads to direct and indirect costs of over 11 billion dollars annually [25]. Understanding whether negative feelings toward the COVID-19 vaccine could translate to resistance to flu vaccines can provide insights to scholars, clinicians, and policymakers.

Researchers used several strategies to evaluate the effect of COVID-19 pandemic on flu vaccine uptake. Cross-sectional studies established a relationship between COVID-19 and flu vaccine uptake [26–28]. A meta-analysis identified a connection between influenza vaccine uptake and COVID-19 vaccine determinants [29]. These conclusions were in line with a study that reported an increase in negative social media posts about flu vaccines since the outbreak of COVID-19 [30].

Longitudinal studies mapped people’s attitudes toward flu vaccination over time. One study conducted in Finland reported an increase in positive attitudes toward flu vaccination following the outbreak of COVID-19 [31]. A panel study of college students conducted in the United States concluded that the COVID-19 pandemic lowered flu vaccine hesitancy [32]. However, the extent of the negative spillover might be uneven and exist only among some groups, such as self-identified Republicans. During the COVID-19 pandemic, the intention to receive a flu vaccination increased among Democrats, but decreased for Republicans [33].

In our study, we utilized a survey experiment to evaluate whether attitudes toward COVID-19 vaccination affect willingness to receive a flu vaccine. We followed the recommendation by Motta to use the experimental method to uncover the presence of COVID-19 vaccine spillover in other health decisions [22]. In experimental studies, participants are randomly assigned to experimental and control groups, and this research design can facilitate a more direct evaluation of whether people’s attitudes toward COVID-19 vaccination will also affect their attitudes toward flu vaccination. The experimental approach is well-suited to evaluate whether the negative spillover will be stronger among groups with negative attitudes toward COVID-19 vaccines, such as Republicans.

We add to the existing experimental studies that explored how the COVID-19 pandemic shaped people’s attitudes toward flu vaccination and other protective behaviors [34–36]. By examining the differences between political parties, we follow the suggestions of Pacheco et al. to understand the politicization of public health [37]. The findings will be relevant to public health officials and clinicians. It’s possible that when encouraging flu vaccination, it might be preferable not to mention COVID-19 vaccination. The results could help to develop more effective communication strategies to encourage annual flu vaccine uptake.

## Methods

### Ethics statement

The authors received approval from the Program Director of Research Integrity and Compliance at South Dakota State University (Approval number: IRB-2024-19) prior to conducting the study. All participants provided written informed consent through the survey instrument.

### Experimental design

The experiment was preregistered and was embedded in an original survey fielded by the authors (https://osf.io/u4qrs?view_only=351ce48173414c988784fe18ec168a1c). Immediately after answering demographic questions and an attention check question, participants were randomly assigned into one of three groups—a control group and two treatment groups. Participants in the first treatment group read a message about the benefits of receiving a flu vaccination. Respondents in the second experimental group read the same message about the benefits of flu vaccination and received information that flu and COVID-19 vaccines could be received simultaneously. The text used in the two treatment conditions was taken nearly verbatim from the CDC website. The participants in the control group did not receive any additional information about flu or COVID-19 vaccines. Immediately afterwards, participants in the two experimental groups received a manipulation check question. Then, all participants answered identical questions about their willingness to receive a flu vaccination and their beliefs in the safety of flu vaccines. We provided the full text of treatment messages (S1 Text) and a flow diagram of the study in the Appendix (S1 Fig).

### Data

Participants in the study were selected through registration based sampling, a commonly used sampling method in the study of mass attitudes [38]. For recruitment, we sampled from the voter data file of all registered voters in the state of South Dakota, obtained from the Secretary of State’s office. We randomly selected 15,600 people from the list who then received an invitation letter to complete an online survey on the QuestionPro survey platform. Each letter included the URL address, as well as a QR code to access the survey site, and a unique six-digit code to open it. Data were collected from April 18^th^ to 29^th^, 2024. The response rate was 4.8%, which is in line with similar surveys using the same recruitment method [38,39]. No financial reward was provided to the participants.

The authors received approval from the Program Director of Research Integrity and Compliance at South Dakota State University (Approval number: IRB-2024-19) prior to conducting the study. All participants provided informed consent through the survey instrument.

### Measures

We used a single question to measure willingness to receive a flu vaccination (1 = very unlikely to 5 = very likely) and a single question to measure belief in the safety of the flu vaccine (1 = not at all safe to 4 = very safe). Scholars previously used these questions to measure attitudes toward flu vaccination [33]. The survey also included questions measuring age (in years), gender (1 = male), ethnicity (1 = white), education (1 = some high school or less to 6 = post-graduate degree), flu vaccination status (1 = received flu vaccination during the last vaccination season, 2 = did not receive flu vaccination during the last vaccination season), COVID-19 vaccination status (1 = Not vaccinated, 2 = Vaccinated, 3 = Vaccinated and boosted, 4 = Vaccinated and boosted within the past 12 months), trust in government (1 = Always to 5 = Never), and partisan self-identification (1 = Democrat, 2 = Independent, 3 = Republican). The choice and the wording of these questions was based on similar studies [26]. An attention check was also included in the survey (S2 Text).

### Analysis

We first described the data. Afterward, we weighted the data based on age, gender, flu vaccination status, and partisan self-identification. Only those participants who passed the attention and manipulation checks were kept in the analysis.

We then conducted difference of means tests (using t-tests) between the two treatment groups and the control group, with likelihood of receiving the flu vaccines and belief in flu vaccine safety as dependent variables. Since the extent literature suggests the spillover effect might be driven in part by partisanship, we also conducted sub-group analysis using the same difference of means tests between Democrats and Republicans on both dependent variables.

Finally, we estimated ordered logistic regression models with likelihood of receiving the flu vaccines and flu safety as dependent variables. The two treatment conditions, age, gender, education, flu vaccination status, COVID-19 vaccination status, trust in government, and political partisanship were the independent variables. We did not include a racial indicator given the extreme homogeneity of the sample. Two models were estimated across the entire sample and four models estimated in their respective partisan subgroups.

All models were weighted. The reported results were based on two-tailed significance tests and 95% level confidence interval. We used case-wise deletion to handle incomplete data. All analysis was conducted on Stata 17 [40].

## Results

Overall, we received 727 responses, with the 591 who passed both the attention check and manipulation check retained for analysis. The average age was 58 years (SD = 16.3), 53% of participants were male, 61% received at least a 4-year degree, 66% received a flu vaccine during the last vaccination season, and 42% received a COVID-19 booster within the last 12 months. Over 29% of participants identified as Democrats, 29% as Independents, and 42% as Republicans. Almost 78% of self-identified Democrats received a COVID-19 booster within the past 12 months, 46% received a booster, 5% completed the initial series, and 2% were not vaccinated. About 19% of self-identified Republicans received a COVID-19 booster within the past 12 months, 22% received a booster, 30% completed the initial series, and 29% were not vaccinated. Fewer than 9% of respondents answered that they never trusted the government to do what is right, 46% trusted the government some of the time, 21% half of the time, 24% most of the time, and less than 1% always trusted the government.

Regarding the likelihood of receiving a flu vaccine in the coming year (range 1–5), there was no statistically significant difference between the control group (mean = 3.6) and the flu and COVID-19 treatment group (mean = 3.6). There was a small, yet statistically significant difference between the control and flu treatment group (mean = 3.2 and p = 0.038). Regarding the safety of the vaccine (range 1–5), The means of the control group, the flu treatment group, and the COVID-19 and flu treatment groups were 3.4, 3.5, and 3.5, respectively; with no statistically significant differences. The results of the partisan sub-group analysis are presented in Tables 1 and 2. While there are distinct partisan differences on both the likelihood of vaccine uptake and the safety of the flu vaccine, we did not observe statistically significant differences within the partisan sub-groups.

**Table 1:** Likelihood of flu vaccination by partisan identification.

	Control	Flu Prime	Covid and Flu Prime
Democrats	4.5	4.6	4.1
Republicans	3.1	2.8	3.3

**Table 2:** Safety of flu vaccination by partisan identification.

	Control	Flu Prime	Covid and Flu Prime
Democrats	3.9	3.9	3.9
Republicans	3.2	3.3	3.3

The results of ordered logistic regression models are displayed in Table 3. The left-hand column showed results of a model with the likelihood of receiving flu vaccination as the dependent variable. The indicators for each experimental group failed to reach statistical significance relative to the control group (omitted category) in each model. Despite relatively strong McFadden’s “pseudo r-square” values that suggested well specified models, the effects of the primes proved to be null. Flu vaccination status, COVID-19 vaccination status, and trust in government were positively and significantly associated with the willingness to receive flu vaccination. The correlations with other variables were not statistically significant.

**Table 3:** Ordered logistic regression results.

	Likelihood of Receiving Flu Vaccination	Belief about Safety of Flu Vaccination
Flu Prime	0.616	1.578
	(0.373 - 1.015)	(0.821 - 3.032)
Flu & COVID Prime	1.012	1.333
	(0.594 - 1.724)	(0.707- 2.511)
Age	0.985	0.98
	(0.971 - 0.999)	(0.966 - 0.994)
Male	1.384	1.378
	(0.888 - 2.159)	(0.805 - 2.539)
Education	1.011	1.073
	(8.844 - 1.211)	(0.889 - 1.293)
Partisan Identification	1.135	0.841
	(0.835 - 1.544)	(0.574 - 1.231)
Flu Vaccination Status	44.073	7.333
	(23.403 - 82.999)	(4.051 - 13.275)
COVID Vaccination Status	2.007	3.358
	(1.528 - 2.636)	(2.413 - 4.672)
Trust in Government	1.473	1.945
	(1.149 - 1.889)	(1.360 - 2.780)
Number of Respondents	539	539
McFadden’s R-Square	0.338	0.366

Cell entries are odds ratios with 95% CIs in parentheses.

The right-hand column of Table 3 showed the results of regression analysis with the attitudes on the safety of the flu vaccine as dependent variable. The results were very similar to the first model. The associations between the experimental group indicators and the dependent variable were not statistically significant. The correlations between flu vaccination status, COVID-19 vaccination status, and trust in government were statistically significant, while all other associations were not significant.

That output presented in the supplementary material modeled Democrats and Republicans separately on both dependent variables (S1 Table). The results were very similar and none of the experimental treatment indicators were statistically significant.

## Discussion

We conducted a preregistered survey experiment to examine whether messages endorsing flu vaccination that also contained COVID-19 vaccine content affected people’s attitudes toward flu vaccination. Overall, we found that our vignettes had little effect on people’s attitudes toward flu vaccine. Those attitudes were primarily driven by peoples’ current flu vaccination status. Our findings will be of interest to both scholars and practitioners of public health.

Vaccination became a polarizing and emotionally charged issue during the COVID-19 pandemic. Some people developed negative attitudes toward COVID-19 vaccination. Experts expressed concern that these negative attitudes toward COVID-19 vaccination might spill over to attitudes toward other vaccines, such as for flu. Studies conducted during the COVID-19 pandemic reported correlations between the uptake of COVID-19 and flu vaccines [28,41,42].

We contributed to the scholarly debate on this topic by conducting a survey experiment. This research design allowed for a more direct test of the spillover effect hypothesis. Nevertheless, we found little evidence of a negative spillover through messaging when it comes to popular attitudes toward flu vaccination. Had a spillover effect been present, we would see evidence in a few separate points in our statistical tests. First, the experimental prime that paired the statement on flu vaccination safety with a message that COVID-19 vaccine could be safely taken with it, failed to reach any conventional level of statistical significance in either the future uptake variable or safety variable. Additionally, we did not uncover any evidence even among self-identified Republicans where we expected the spillover effect to be strongest. Despite abundant evidence that COVID-19 attitudes are significantly influenced by patients’ political attitudes, that politicization did not carry over to flu vaccine attitudes – even when the two vaccines were paired in the vignette. Should a spillover effect be real, we would expect the paired message to move attitudes amongst Republicans in both the future uptake and belief in flu vaccine safety models.

We also found that partisan self-identification did not correlate with attitudes toward flu vaccination. During the COVID-19 pandemic, COVID-19 vaccination became a politically polarizing topic and peoples’ attitudes toward COVID-19 vaccines were strongly shaped by their partisan self-identification. Scholars expressed concern that those who identified with the Republican Party were more likely to develop negative attitudes toward other vaccines [22,43]. The findings in the extant research have been mixed. One recent study reported that Republicans were more likely to receive a flu vaccine than Democrats, however, their opposition to flu vaccine uptake was mediated by lowered COVID-19 vaccine uptake [26].

Our findings suggested that flu vaccine, perhaps because it has been offered for a long time, was immune to the negative emotions that were often associated with COVID-19 vaccine. Flu vaccine is an established vaccination and was also a less attractive target for politicians compared to COVID-19 vaccine, which was novel and thus prone to politicization. Perhaps for these reasons, in both models, prior flu vaccination was most important in determining attitudes toward receiving the flu vaccine and flu vaccine safety.

The study was limited by the fact that all participants came from one state, which is more ethnically homogenous and more socially and politically conservative than the rest of the United States. Compared to other work exploring attitudes toward flu and COVID-19 co-vaccination, such as an Italian study based on a nationally-representative survey [44], generalization of our findings must be made with caution. We relied on the list of registered voters to recruit participants, and we were not able to invite those residents of South Dakota who were not registered to vote. Racial and ethnic minorities were also underrepresented in our study population with 96% of the sample identifying as white. Though our response rate is in line with similar studies using the same participant recruitment method, it is lower than other recruitment methods, and could increase the potential for selection bias. Future research should expand the study to a more nationally representative sample, where the attitudes of racial and ethnic minorities can be properly assessed. We also encourage scholars to explore how flu vaccination encouragements from trusted community leaders, such as clergy, can affect people’s attitudes toward receiving flu vaccination. This study only examined people’s attitudes toward flu vaccination. We invite scholars to explore how COVID-19 pandemic might have affected people’s attitudes toward other vaccines and health treatments. Finally, research that examines actual medical outcomes – and not just attitudes and intentions - would be most welcome. Ideally, this could be studied in a clinical setting where similar primes could be used by health care providers in patient consultations where vaccination decisions could be confirmed.

The findings also have implications for public health officials and clinicians. Experts have long acknowledged the challenges associated with flu vaccination communication [45,46]. The politicization of vaccination during the COVID-19 pandemic created challenges for public health communication [47]. Given the sensitive and emotionally charged nature of COVID-19 vaccines, healthcare workers might be reluctant to mention them when discussing other vaccines or health treatments. Providers might be concerned that just mentioning COVID-19 vaccination could trigger negative emotions and might make the patients less receptive to recommendations regarding other vaccines or health treatments. Our results suggest that healthcare practitioners should not shy away from mentioning COVID vaccination along with flu vaccination, even in groups with higher COVID-19 vaccine hesitancy. This finding also holds among Republicans, a group with lower COVID-19 vaccine uptake and more negative attitudes towards COVID-19 vaccination. The findings of our study have similar implications for public health officials. The results suggest that mentioning COVID-19 vaccines together with flu vaccines in promotion messages did not diminish people’s interest in receiving a flu vaccination. A simultaneous promotion of both vaccines may help streamline resources without a backlash against flu vaccination as some have feared.

## Conclusions

In this study, we conducted a survey experiment to investigate how the framing of messages affected people’s attitudes toward flu vaccination. We found that mentioning COVID-19 vaccine in a message encouraging flu vaccination did not negatively affect people’s willingness to receive a flu vaccination or their belief in the safety of flu vaccines. We discovered the same among self-identified Republicans, a group that developed negative attitudes toward COVID-19 vaccination and reported lower COVID-19 vaccine uptake. Following the outbreak of COVID-19, scholars expressed a concern that these negative attitudes will affect attitudes toward other vaccines. Our results suggested that flu vaccination attitudes were not negatively affected by mentions of COVID-19 vaccination. Public health workers need not shy away from discussing flu and COVID-19 vaccinations together, fearing that mentioning the latter will negatively affect people’s flu vaccine attitudes.

## Supporting information

S1 Fig Flow Diagram. (DOCX)

S1 Table Ordered Logistic Regression Models by Partisan Self-Identification. (DOCX)

S1 Text Experimental Vignettes. (DOCX)

S2 Text Survey Questions. (DOCX)
