# Supplementary material for: No negative effect of mentioning COVID-19 vaccine in influenza vaccine encouragements: Evidence from a survey experiment
Source: PLOS Glob Public Health. 2025 Sep 10;5(9):e0005180. doi: 10.1371/journal.pgph.0005180 (PMC12422471; doi:10.1371/journal.pgph.0005180)
Supplement: S1 Fig — (DOCX) [file pgph.0005180.s001.docx]

**S1 Fig: Flow Diagram**

Invitation letters sent

(n = 15,650)

Responses received

(n = 727)

Passed attention check

(n = 670)

Treatment Group 2

(n = 225)

Control Group

(n = 224)

Treatment Group 1

(n = 218)

#

Analyzed (n = 174)

Those who failed manipulation check were excluded from analysis.

Analyzed (n = 193)

Those who failed manipulation check were excluded from analysis.

Analyzed (n = 224)
