# Supplementary material for: No negative effect of mentioning COVID-19 vaccine in influenza vaccine encouragements: Evidence from a survey experiment
Source: PLOS Glob Public Health. 2025 Sep 10;5(9):e0005180. doi: 10.1371/journal.pgph.0005180 (PMC12422471; doi:10.1371/journal.pgph.0005180)
Supplement: S1 Table — (DOCX) [file pgph.0005180.s002.docx]

**S1 Text: Experimental Vignettes**

Flu Vaccine Treatment

Please examine the following information about seasonal flu vaccination carefully.

Why should people get vaccinated against flu?

Flu is a potentially serious disease that can lead to hospitalization and sometimes even death. Every flu season is different, and flu can affect people differently, but during typical flu seasons, millions of people get flu, hundreds of thousands of people are hospitalized and thousands to tens of thousands of people die from flu-related causes.

An annual seasonal flu vaccine is the best way to help reduce the risk of getting flu and any of its potentially serious complications. Vaccination has been shown to have many benefits including reducing the risk of flu illnesses, hospitalizations and even the risk of flu-related death. While some people who get a flu vaccine may still get sick with flu, flu vaccination has been shown in several studies to reduce severity of illness.

Flu and Covid Vaccine Treatment

Please examine the following information about seasonal flu vaccination carefully.

Why should people get vaccinated against flu?

Flu is a potentially serious disease that can lead to hospitalization and sometimes even death. Every flu season is different, and flu can affect people differently, but during typical flu seasons, millions of people get flu, hundreds of thousands of people are hospitalized and thousands to tens of thousands of people die from flu-related causes.

An annual seasonal flu vaccine is the best way to help reduce the risk of getting flu and any of its potentially serious complications. Vaccination has been shown to have many benefits including reducing the risk of flu illnesses, hospitalizations and even the risk of flu-related death. While some people who get a flu vaccine may still get sick with flu, flu vaccination has been shown in several studies to reduce severity of illness.

Can flu vaccines and COVID-19 vaccines be given at the same time?

Yes, getting a flu vaccine and COVID-19 vaccine at the same visit is recommended if you are eligible and the timing for each vaccine is right.

Is it safe to get a flu vaccine and a COVID-19 vaccine at the same time?

Studies conducted throughout the COVID-19 pandemic supported the safety of getting a flu vaccine and COVID-19 vaccine at the same visit.
