# Supplementary material for: No negative effect of mentioning COVID-19 vaccine in influenza vaccine encouragements: Evidence from a survey experiment
Source: PLOS Glob Public Health. 2025 Sep 10;5(9):e0005180. doi: 10.1371/journal.pgph.0005180 (PMC12422471; doi:10.1371/journal.pgph.0005180)
Supplement: S2 Text — (DOCX) [file pgph.0005180.s004.docx]

**S2 Text: Survey Questions**

Likelihood of flu vaccine uptake

"How likely is it that you will get a flu vaccine for the 2024-2025 season?" (1=Very unlikely, 2=Unlikely, 3=Neither likely nor unlikely, 4=Likely, 5=Very likely)

Flu vaccine safety

"How safe do you believe the seasonal flu vaccine is for most people?" (1=Not at all safe, 2=Not very safe, 3=Somewhat safe, 4=Very safe)

Age

"What was your age on your last birthday?"

Gender

"What is your gender?" (1= male, 2= female, 3=other)

Education

"What is the highest level of education you have completed?” (1= some high school - 6= post-graduate degree)

COVID-19 vaccination status

"What is your COVID-19 vaccination status?" (1= Not vaccinated, 2= Vaccinated, 3= Vaccinated and boosted, 4= Vaccinated and boosted within the past 12 months)

Flu vaccination status

"Did you receive a flu vaccine during the 2023-2024 flu season?" (1=yes, 2=no)

Political party identification

"Generally speaking, do you think of yourself as a Democrat, Republican, an Independent, or something else?" (1= Democrat, 2= Republican, 3= Independent, 4= Something else)

Trust in government

"How often can you trust the government to do what is right?" (1= Always, 2= Most of the time, 3= Half of the time, 4= Some of the time, 5= Never)

Attention check question

“Researchers found that participants sometimes answer survey questions without reading them carefully, which could affect data quality. Please select strongly agree to show that you are paying attention to this question.” (1= Strongly agree, 2= Agree, 3= Disagree, 4= Strongly disagree)

Manipulation check question

“Do you recall any of the following vaccines on the previous screen? Select all that apply.” (1= "Flu vaccine" 2= "Covid vaccine" 3= "RSV vaccine")

**Variable Transformations**

Gender

"Other" answers were removed.

Manipulation check

Correct answers ("Flu vaccine" only in the Flu Vaccine Treatment, and "Flu vaccine" and "Covid vaccine" only in the Flu and Covid Vaccine Treatment ) were coded as 1 and other answers were coded as 0.

Attention check

“Strongly agree” answers were coded as 1 and other answers 0.

Political party identification

"Other" answers were removed.
